# Exploration of Association Between Respiratory Vaccinations With Infection and Mortality Rates of COVID-19

**DOI:** 10.1017/dmp.2021.47

**Published:** 2021-02-16

**Authors:** Deldar Morad Abdulah, Alan Bapeer Hassan

**Affiliations:** 1 Community and Maternity Health Unit, College of Nursing, University of Duhok, Duhok, Iraqi Kurdistan; 2 Basic Sciences Unit, College of Nursing, University of Duhok, Dohuk, Iraqi Kurdistan

**Keywords:** respiratory infections, COVID-19, disease burden, routine immunization, nonspecific immunity

## Abstract

**Objective:**

Respiratory disease vaccines may affect coronavirus disease 2019 (COVID-19) - associated infection and mortality rates due to vaccine nonspecific effects against viral infections. We compared the infection and mortality rates in relation to COVID-19 between countries with and without universal respiratory disease vaccine policies.

**Methods:**

In this ecological study, 186 countries with COVID-19 statistics from the World Health Organization (WHO) were included.

**Results:**

The study found that countries with universal BCG (bacillus Calmette Guérin) vaccine had significantly lower total infection and mortality rates, 0.2979 and 0.0077 versus 3.7445, and 0.0957/1000 people and confirmed cases (*P* < 0.001). The countries with universal pneumococcal vaccine (PCV), including PCV1, PCV2, and PCV3 vaccines, had significantly higher total mortality, 0.0111 versus 0.0080, respectively (*P* = 0.032). Higher income was associated with increasing total infection and mortality rates. Whereas, BCG vaccination was associated with a lower total mortality rate only (*P* = 0.030). The high-income countries were more likely to not receive universal BCG and receive second dose of meningococcal conjugate vaccine (MCV2) and third dose of PCV3 vaccination coverage. The severe acute respiratory syndrome coronavirus 2 (SARS-CoV-2) infection rates increased with increasing years of the second dose of measles-containing vaccine (*P* = 0.026) and pneumococcal conjugate third dose (PCV3).

**Conclusions:**

This study suggests that BCG vaccination could reduce the infection caused by COVID-19, and MCV2 vaccine years increases the total infection rate. This study identified high economic characteristics and not having universal BCG coverage as the independent risk factors of mortality by multivariate analysis.

The recent coronavirus outbreak announced in China has spread dramatically to several countries across the world.^1,2^ Globally, by October 15, 2020, there have been 38,394,169 confirmed cases of coronavirus disease 2019 (COVID-19), including 1,089,047 deaths, reported to the World Health Organization (WHO).^3^ Currently, the COVID-19 pandemic has become a serious threat to the world. Coronaviruses are a group of highly diverse, enveloped, positive-sense, single-stranded RNA viruses.^4^ This family of viruses can infect the respiratory, enteric, hepatic, and neurological systems and cause a variety of diseases in humans.^4,5^

The meta-analysis of clinical, laboratory, and imaging features of COVID-19 showed that fever (88.7%), cough (57.6%), and dyspnea (45.6%) are the most prevalent manifestations. In addition, 20.3% of the patients require intensive care unit care, 32.8% present with acute respiratory distress syndrome, and 6.2% with shock. The case fatality rate of hospitalized patients is 13.9%.^6^ The evidence has shown person-to-person transmission among clusters of infected family members and medical workers.^7,8^

While endemic human coronaviruses (OC43, 229E, NL63, and HKU1) tend to cause mild respiratory illness, the three epidemic species (severe acute respiratory syndrome coronavirus 1 [SARS-CoV-1], Middle East respiratory syndrome coronavirus [MERS-CoV], and severe acute respiratory syndrome coronavirus 2 [SARS-CoV-2]) are capable of causing severe disease in humans.^9^ According to some investigations and clinical data of patients diagnosed with COVID-19 in the early outbreak, the reproductive number (R_0_) of this disease is between 2.20 and 3.58.^10,11^ In other words, each patient transmits the virus to between 2 and 4 other persons. The mean incubation period is 5 d (range, 1-14 d), and 95% of detected patients experience disease symptoms within 12.5 d of exposure.^10,12^

WHO declared SARS-CoV-2 infection a public health emergency of international concern on January 30, 2020, and escalated this declaration to that of the pandemic on March 11, 2020. Currently, there is no effective therapy or vaccine for this disease. However, observational evidence suggests that the bacillus Calmette Guérin (BCG) vaccine may impact rates of SARS-CoV-2 infection and mortality. For example, by April 30, 2020, a total of 71 days after the detection of the first confirmed COVID-19 case in Iraq, a country with routine BCG vaccination, there were 2085 confirmed COVID-19 cases and 93 deaths (crude mortality rate: 4.6%). However, in Italy, where BCG is not routinely administered, at day 71 after detection of the first COVID-19 case there were 92,472 confirmed cases and 10,023 deaths (crude mortality rate: 10.8%).^3^ Similar disparities in case fatality rate between countries with routine BCG coverage and those that do not use BCG routinely have been described elsewhere.^3^

Viral respiratory infections are responsible for considerable morbidity and mortality in infants and young children as well as in at-risk adults and the elderly.^13^ Respiratory syncytial virus (RSV) is the single most important causative factor of lower respiratory tract illness in infants <1 y of age. Moreover, RSV causes repeat infections and remarkable disease throughout life. Young children, immunocompromised individuals, persons with compromised pulmonary or cardiac systems, and the elderly are at significant risk of infection.^14^

Moorlag et al. (2019)^15^ reviewed the nonspecific effects of BCG against viral infections. They reported several epidemiological, immunological, and clinical studies that suggest that BCG vaccination results in immunological responses that lead to decreased rates of infection and mortality. However, studies that show a protective effect of BCG against viral infections, including influenza and herpes simplex virus, are largely from experiments conducted on mice.^15^ Furthermore, the effectiveness of BCG and other vaccines that target respiratory pathogens has not been evaluated as yet on COVID-19.

We compared the crude infection and mortality rates of COVID-19 between countries with and without universal respiratory vaccination policies. Also, we aimed to examine the link between infection and mortality rates of COVID-19 with universal coverage policies of respiratory diseases across the globe for the period 1980-2018. We hypothesized that the infection rate of the disease could be linked to the age at vaccination or vaccine years for respiratory diseases. In this study, we defined universal vaccine coverage as vaccine coverage at the national level.

## Methods

### Study Design and Sampling

In this ecological study, we included countries that had or had not implemented vaccination policies that covered respiratory infections^16^ and had data available on COVID-19 infection and mortality through the WHO website.^3^ The countries with coronavirus disease statistics from the WHO were consecutively checked for the national respiratory vaccine policies for the period 1980-2018. The information on cases and deaths of the countries was extracted from the WHO Coronavirus Disease (COVID-19) Dashboard by May 29, 2020.^3^

## Inclusions and Exclusion Criteria

Countries and states met eligibility for inclusion in this study if data were available from the WHO coronavirus disease situation dashboard by May 29, 2020.^3^ Accordingly, the following countries were excluded from the analysis; Marshall Islands, Nauru, Palau, and Tuvalu due to not having statistics on the COVID-19 outbreak.

Immunization schedules of countries that were included in the first step were obtained from the WHO website and were excluded from further analysis if immunization statistics were unavailable.^16^ Accordingly, the following countries were not included due to not having an immunization schedule on the WHO website: Anguilla, Aruba, Bermuda, British Virgin Islands, Cayman Islands, Curacao, Dimond Princess, Faroe Islands, French Guiana, French Polynesia, Gibraltar, Greenland, Guadeloupe, Guam, Guernsey, Isle of Man, Jersey, Martinique, Montserrat, Myotee, New Caledonia, Northern Mariana Islands, Palestine, Puerto Rico, Reunion, Saint Barthelemy, Saint Martin, Saint Maarten, Turks and Caicos Islands, and the Virgin Islands.

Populations of the included countries were extracted from the United Nations Statistics Division.^17^ The World Population Prospects 2019 was used to obtain the male and female populations. As population denominator data were not available for Dominica, Kosovo, Saint Kitts, and Nevis, these countries/territories were excluded from further analysis.^17^

The types of health systems of the countries were drawn from the “Social Security Programs Throughout the World” published by The United States Social Security Administration (SSA).^18^ The following countries did not have health system statistics from the SSA website: Afghanistan; Bosnia and Herzegovina; Comoros; Costa Rica; Côte d’Ivoire; Croatia; Cuba; Cyprus; Czechia; Eritrea; Democratic People’s Republic of Korea; Maldives; Mongolia; Montenegro; North Macedonia; Somalia; South Sudan; Timor-Leste; Tonga, and the United Arab Emirates. We used the following 5 categories of the health system: (1) social assistance system; (2) social insurance, and social assistance system; (3) social insurance; (4) universal, social insurance, and social assistance system; and (5) mandatory individual account system.^18^

The economic status was categorized as the following 4 categories: (1) low-income economies, (2) lower middle-income economies, (3) upper middle-income economies, and (4) high-income economies, based on the World Bank classification.^19^

The following respiratory vaccines were included in this study: BCG, DTP1 (first dose of diphtheria toxoid, tetanus toxoid, and pertussis), DTP3 (third dose of diphtheria toxoid, tetanus toxoid, and pertussis), MCV1 (measles-containing vaccine first dose), MCV2 (measles-containing vaccine second dose), PCV1 (pneumococcal conjugate vaccine first dose), PCV2 (pneumococcal conjugate vaccine second dose), and PCV3 (pneumococcal conjugate vaccine third dose).^16^

In this study, the universal coverage between 1980 and 2018 was used for 1980-2018 for BCG; 2000-2018 for DTP1 and DTP3; 1980-2018 for MCV1; 1999-2018 for MCV2; and 2008-2018 for PCV1, PCV2, and PCV3. The cutoff periods of the vaccines are different because some of these vaccines were started and developed at different times.

### Statistical Analyses

The prevalence of COVID-19 in countries with national vaccine coverage data was determined in absolute numbers and as a proportion of the total population (expressed as infection rate per 1000 population). The number of COVID-19 fatalities was divided by the number of confirmed cases and multiplied by 1000 to obtain the crude mortality rate. The normality of the data was examined using histograms and box plots. COVID-19 infection and mortality rates were summarized as medians and interquartile ranges (IQRs) after potential outliers were accounted for. The natural logarithm (LN) transformation technique was used to obtain the normal distribution for age at vaccination and vaccine years.

The differences in COVID-19 infection and mortality rates between countries with and without national vaccination policies were presented in cumulative distribution function (CDF) graphs. Differences in the weighted averages of COVID-19 infection and mortality rates were examined using the Mann-Whitney *U*-test. The differences in weighted averages of the COVID-19 infection and mortality rates in countries with different health systems were examined using the Kruskal Wallis test.

COVID-19 infection and mortality rates were logarithmically transformed to obtain normally distributed outcomes. In generalized linear model (GLM) multivariate analysis of covariance (MANCOVA), COVID-19 infection and mortality rates were considered being dependent variables, with the completeness of universal respiratory vaccine years and age at vaccination the controlling factors. The total male and female populations were considered covariates in this analysis. Statistical significance was assumed if *P*-values were <0.05. The association of respiratory vaccination with economic status was examined in Pearson chi-squared tests. All analyses were done using the Statistical Package for Social Sciences (SPSS) (IBM SPSS Statistics for Windows, version 25.0. Armonk, NY: IBM Corp). CDF and Map graphs were generated using JMP 14.3 (SAS Institute Inc., Cary, NC).

### Ethical Perspectives

As this was an analysis of data that are freely available in the public domain, no institutional review board ethics permissions were considered necessary.

## Results

Of the 186 countries included in this study, 149 (81%) had universal BCG coverage. All countries had introduced universal DTP1 and DTP3 vaccination, and only 1 country had not introduced universal MCV1 vaccination. A total of 149 countries had a universal MCV2 vaccination policy, and 145 had universal coverage with PCV1, PCV2, and PCV3. Vaccination coverage and the mean number of years of universal coverage for each of the vaccines are summarized in Table 1.

**Table 1.** Prevalence of using respiratory vaccines in the world between 1980 and 2018

Respiratory vaccines (*n* = 186)	Universal vaccinations no. (%)		Vaccine coverage of countries with universal vaccine coverage Mean (SD)			
	With universal vaccine coverage	Without universal vaccine coverage	Coverage percentage	Range	Years applied	Min-Max
BCG	149 (80.1)	37 (19.9)	87.34 (10.48)	46.75-99.00	24.32 (2.68)	8-26
DTP1	186 (100)	0 (0.0)	91.70 (8.20)	49.50-99.00	16.74 (4.46)	1-19
DTP3	186 (100)	0 (0.0)	84.92 (12.08)	40.54-99.00	24.22 (2.61)	1-19
MCV1	185 (99.5)	1 (0.5)	84.06 (11.99)	42.25-99.00	24.11 (2.57)	5-26
MCV2	149 (80.1)	37 (19.9)	78.31 (19.03)	25.33-98.50	12.22 (5.85)	1-20
PCV1	145 (78.0)	41 (22.0)	86.59 (14.19)	27.00-99.00	6.18 (2.51)	1-11
PCV2	145 (78.0)	41 (22.0)	83.09 (18.65)	7.00-99.00	5.32 (1.87)	1-7
PCV3	145 (78.0)	41 (22.0)	76.80 (21.43)	10.00-99.00	6.57 (2.76)	1-11

**Min**: Minimum; **Max**: Maximum

Median and IQR crude infection rates of COVID-19 (per 1000 people) were 0.4637 and 2.0549 (males), 0.4637 and 2.1712 (females), and 0.4729 and 2.4469 (total population) overall. Additionally, median and IQR crude COVID- 19 mortality rates (per 1000 confirmed cases) were 0.0105 and 0.0524 (males), 0.0105 and 0.0518 (females), and 0.0105 and 0.0521 (total population), respectively, overall by May 29, 2020 (data not shown in tables).

**Figure 1.**
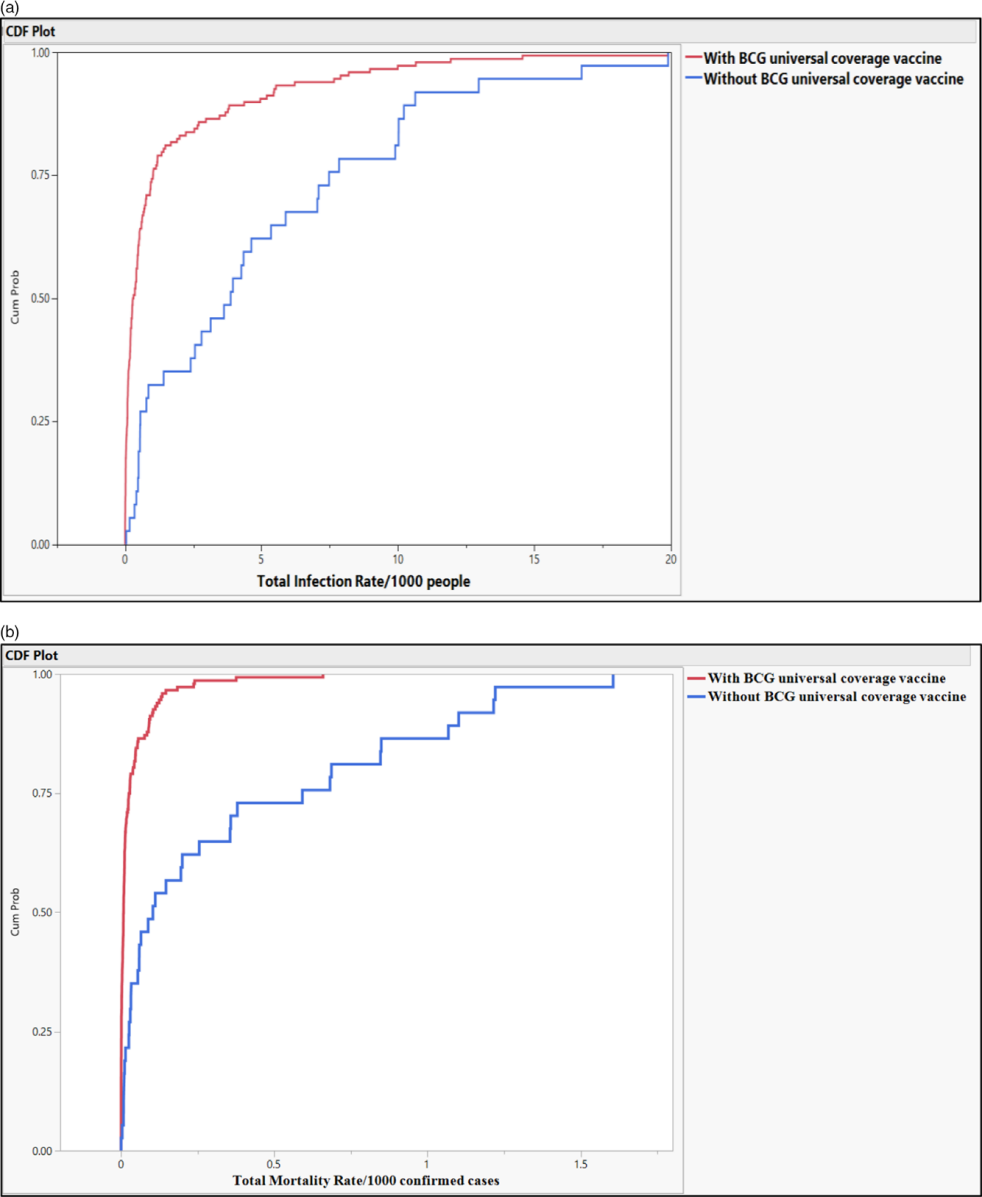
(a-b) Probability of total infection rate of the COVID-19/1000 people and mortality rate of the COVID-19/1000 confirmed cases in countries with and without universal BCG vaccinations.

Countries with universal BCG vaccination had significantly lower infection and mortality rates. The median of total crude infection rates (per 1000 people) was 0.2979 in countries with universal BCG vaccination policies, compared with 3.7445 in countries without universal BCG vaccination. The median of total crude mortality rates (per 1000 confirmed cases) was 0.0077 in countries with universal BCG vaccination policies, compared with 0.0957 in countries without universal BCG vaccination. Countries with universal PCV1, PCV2, and PCV3 vaccination policies had significantly higher total mortality (per 1000 confirmed cases) compared with those without universal PCV vaccination (0.0111 vs 0.0080; *P* = 0.032). The study did not find a significant difference in the total crude median rates of infection and mortality in countries with and without the MCV2 vaccine (0.4729 and 0.3100 vs 0.0109 and 0.0082, *P* = 0.599 and *P* = 0.773, respectively), see Table 2 and Figure 1.

**Table 2.** Univariate comparison of infection rate of COVID-19/1000 people and mortality rate of COVID-19/1000 confirmed cases in countries with and without universal vaccine coverage

Infection and mortality rates	Groups of countries		*P*-Value
	With universal coverage vaccine (median)	Without universal coverage vaccine (median)	
**BCG**			
Total infection rate/1000 people	0.2979	3.7445	**<0.001**
Total mortality rate/1000 confirmed cases	0.0077	0.0957	**<0.001**
**MCV2**			
Total infection rate/1000 people	0.4729	0.3100	0.599
Total mortality rate/1000 confirmed cases	0.0109	0.0082	0.773
**PCV1**			
Total infection rate/1000 people	0.4851	0.3200	0.060
Total mortality rate/1000 confirmed cases	0.0111	0.0080	**0.032**
**PCV2**			
Total infection rate/1000 people	0.4851	0.3200	0.060
Total mortality rate/1000 confirmed cases	0.0111	0.0080	**0.032**
**PCV3**			
Total infection rate/1000 people	0.4851	0.3200	0.060
Total mortality rate/1000 confirmed cases	0.0111	0.0080	**0.032**

Note: Mann-Whitney U-test was performed for statistical analyses. The bold numbers show the significant difference.

**Figure 2.**
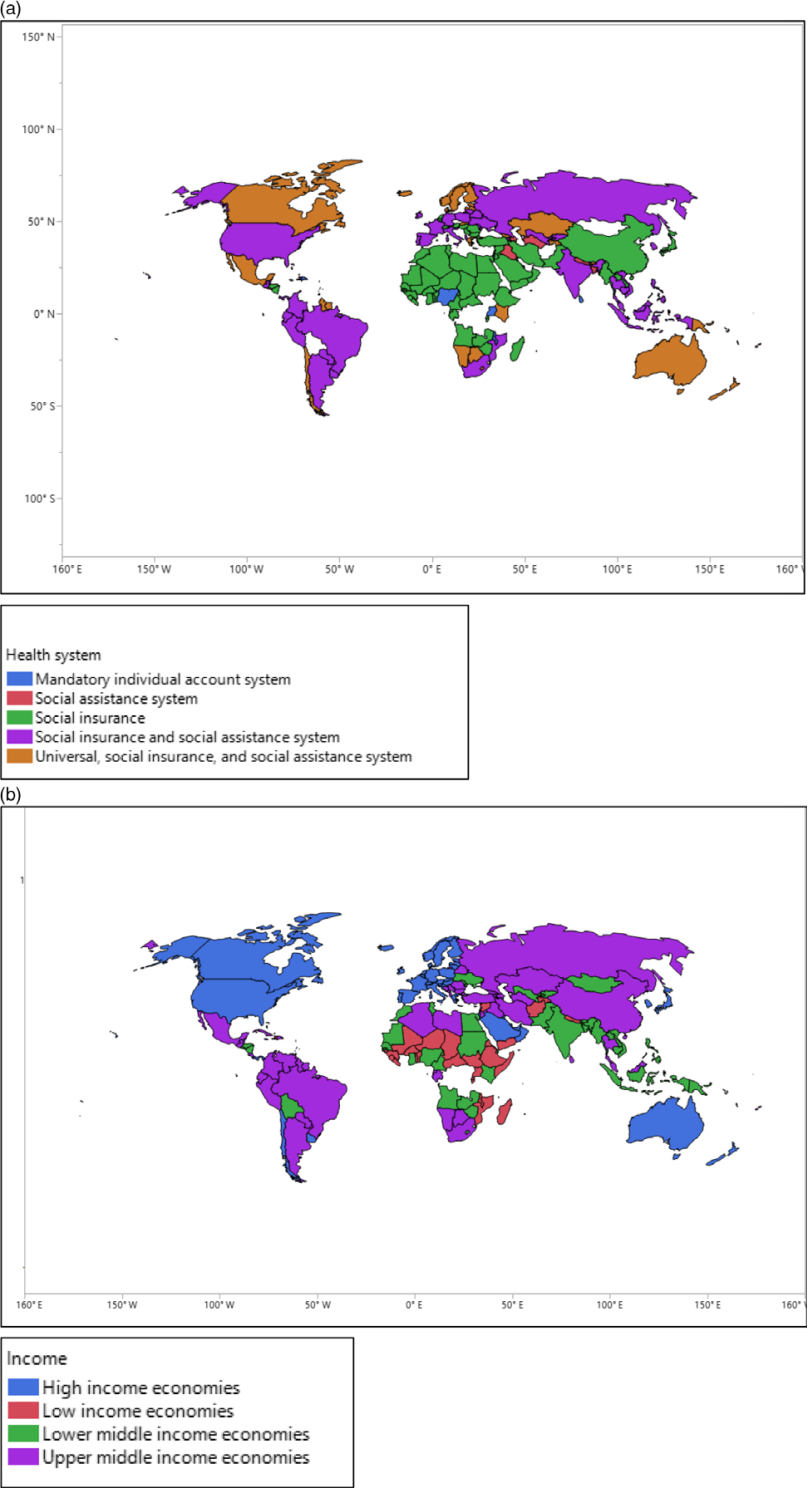
(a,b) The geographic areas of countries with different health systems and economic status.

**Figure 3.**
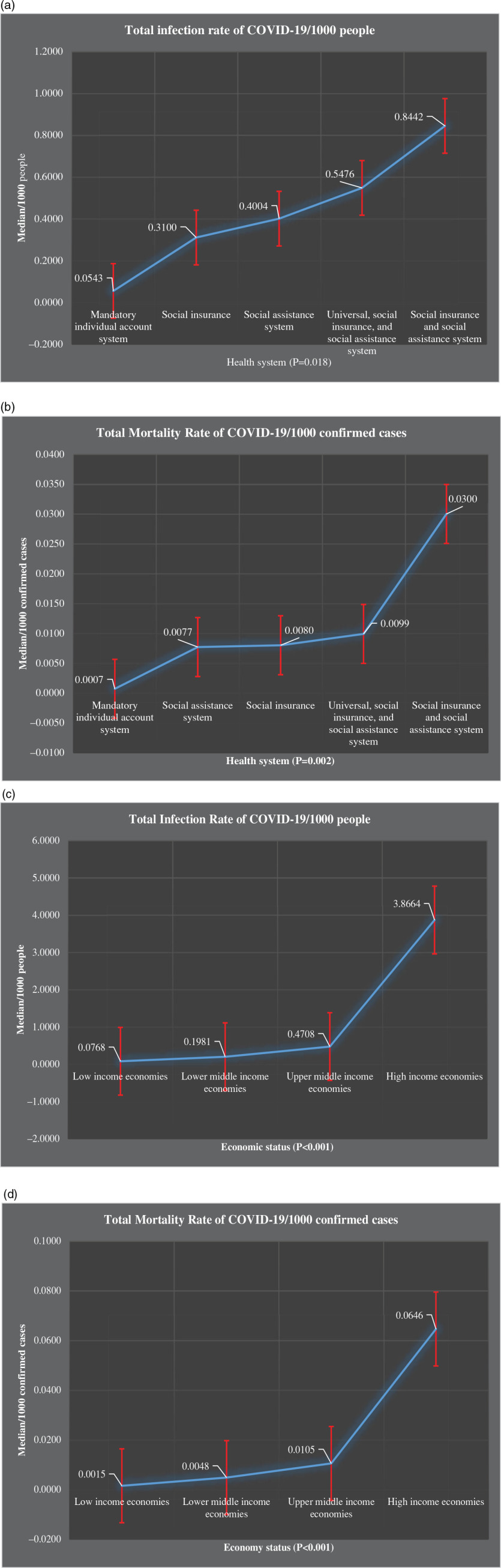
(a-d) Comparison of total infection rate of COVID-19/1000 people and total mortality rate of COVID-19/1000 confirmed cases among countries with different health systems and economic status.

**Figure 4.**
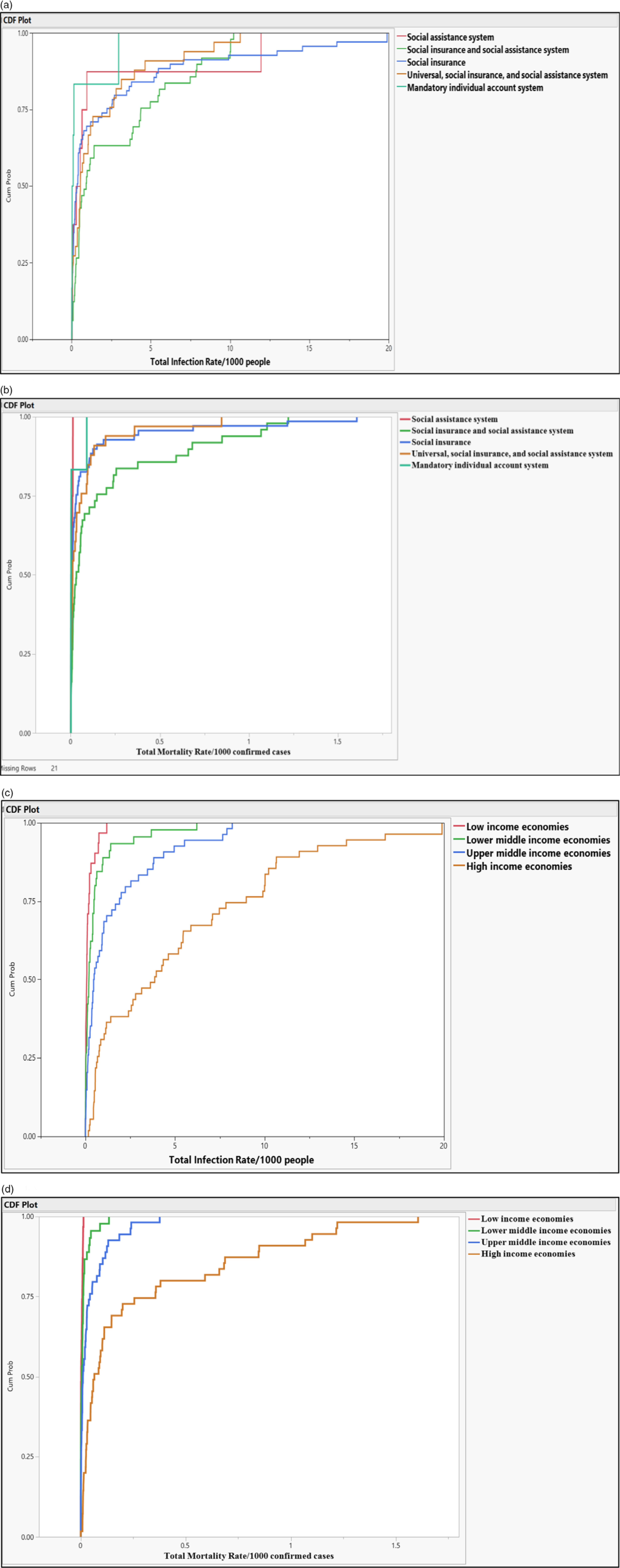
(a-d) Probability of total infection rate of the COVID-19/1000 people and total mortality rate of the COVID-19/1000 confirmed cases in countries with different health system and economy status.

The medians of total infection rates of COVID-19/1000 people were increased from mandatory individual accounts to social insurance and social assistance system, 0.0543, 0.3100, 0.4004, 0.4576, and 0.8442. A similar pattern was found for the total mortality rates of COVID-19/1000 confirmed cases, 0.007, 0.0077, 0.0080, 0.0099, and 0.0300, respectively. Countries with social insurance and social assistance systems had substantially higher infection rates (0.8442 per 1000 people) and mortality rates (0.0300 per 1000 confirmed cases) compared with other countries. In contrast, countries with mandatory individual account systems had significantly lower infection and mortality rates; 0.0534 and 0.0007 per 1000 confirmed cases, respectively. The total infection rate of COVID-19 increased in countries from low income to high income, 0.0768, 0.1981, 0.4708, and 3.8664, respectively. A similar pattern was found in terms of the mortality rate of COVID-19: 0.0015, 0.0048, 0.0105, and 0.0646, respectively. Countries and their health system types are presented in Figures 2-4.

**Figure 5.**
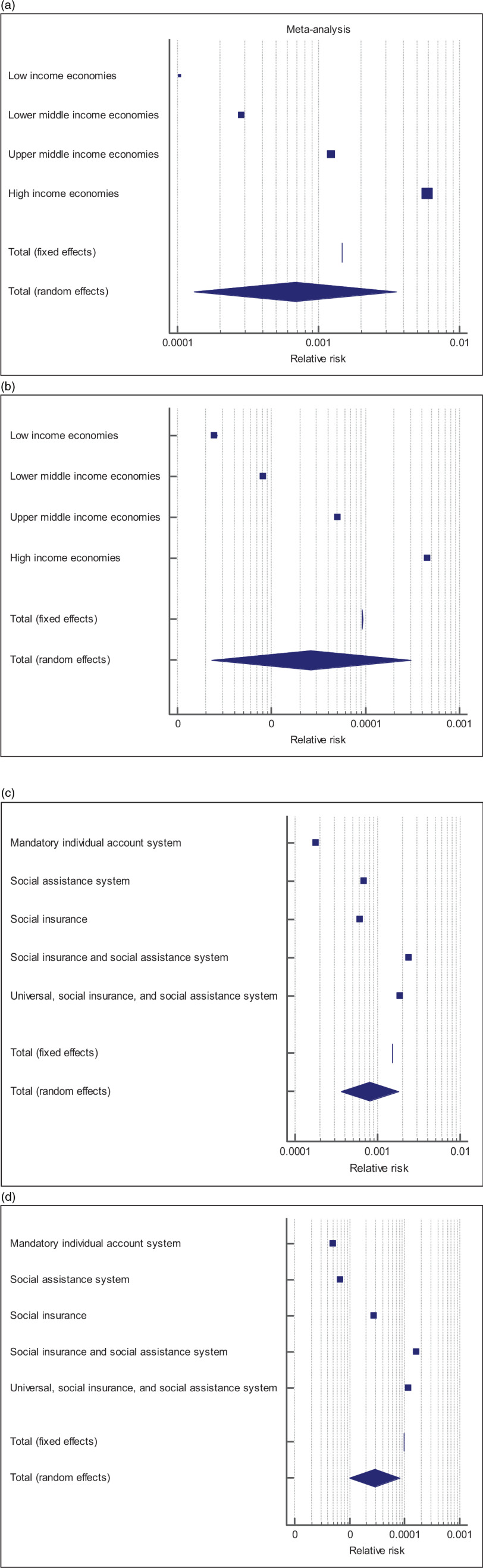
(a-d) Forest plots of relative risk of countries according to health system and economic status.

We performed three sets of multivariate analyses by MANCOVA GLM to determine the role of respiratory vaccination on infection and mortality rate of the COVID-19/1000 confirmed cases. In models that explored the relationship between varying population characteristics and respiratory vaccinations, lower-income status was significantly associated with a lower total crude infection and mortality rates of COVID-19 (*P* < 0.001). Whereas, BCG coverage was significantly associated with a lower total crude mortality rate of COVID-19 only (hazard ratio [HR], 286.837; 95% confidence interval [CI], 284.398-289.317; *P* = 0.030); see Tables 3 and 4 and Figure 5.

**Table 3.** Association of total infection rate of COVID-19/1000 people and total mortality rate of COVID-19/1000 confirmed cases with respiratory vaccinations, age at vaccination, and vaccine years in the world between 1980 and 2018

Controlling factors	Dependent variable			
	Total infection rate/1000 people		Total mortality rate/1000 confirmed cases	
Respiratory vaccine coverage (*n* = 186)	*P*-Value	HR (95% CI)	*P*-value	HR (95% CI)
**Economic characteristics**	**<0.001**		**<0.001**	
Low-income economies				
Lower middle-income economies		2.73 (2.75-2.70)		3.31 (3.46-3.16)
Upper middle-income economies		11.83 (11.84-11.70)		20.28 (21.42-19.20)
High-income economies				
				185.08 (196.14-174.62)
		57.21 (57.67-56.32)		
**Health system**	0.481		0.161	
Mandatory individual account system				
Social assistance system				
Social insurance		3.80 (3.82-3.77)		1.34 (1.35-1.34)
Social insurance and social assistance system		3.41 (3.44-3.36)		5.58 (5.94-5.25)
		13.46 (13.62-13.30)		33.00 (35.19-30.91)
Universal, social insurance, and social assistance system		10.34 (10.45-10.27)		23.40 (24.68-21.97)
**BCG coverage**	0.681		**0.030**	
With universal coverage vaccine				
Without universal coverage vaccine		426.528 (447.221-407.665)		286.837 (284.398-289.317)
**MCV2 coverage**	0.216		0.271	
With universal coverage vaccine				
Without universal coverage vaccine		7.602 (7.596-7.608)		108.994 (108.627-109.364)
**PCV coverage**	0.657		0.485	
With universal coverage vaccine				
Without universal coverage vaccine		7.319 (7.307-7.331)		90.609 (90.080- 91.143)
Male population	0.877		0.230	
Female population	0.897	NA	0.231	NA

Note: Multivariate analysis model was performed for statistical analyses.

The bold numbers shows the significant difference.

Vaccine years: The number of years that the vaccine was applied since it commencement.

The first row was considered the reference in calculating the HR and 95% CI.

Abbreviation: NA, not applicable.

**Table 4.** Association of total infection rate of COVID-19/1000 people with DTP3, MCV2, and PCV3 with adjustment for total population, health system, and economy status

Total infection rate/1000 people	Vaccine years		
	DTP3	MCV2	PCV3
Correlation	−0.068	0.006	0.398
R Squared	0.00462	0.00004	0.15840
Significance (2-tailed)	0.481	0.947	<0.001

The adjustment was made for total population, health system, and economy status.

Regarding age at receiving respiratory vaccines, only lower-income status was significantly associated with a total infection rate of COVID-19 (*P* = 0.003), but not with a total mortality rate of COVID-19 (*P* = 0.098), with no association with age at vaccination for other respiratory vaccines (data not shown in tables). Increasing years of DTP3 universal coverage was significantly associated with decreasing COVID-19 infection rates; *P* = 0.017. Whereas, increasing years of MCV2 (*P* = 0.026) and PCV3 (*P* = 0.024) were associated with increasing total infection rate of COVID-19 (this multivariate analysis was not shown in the tables).

**Figure 6.**
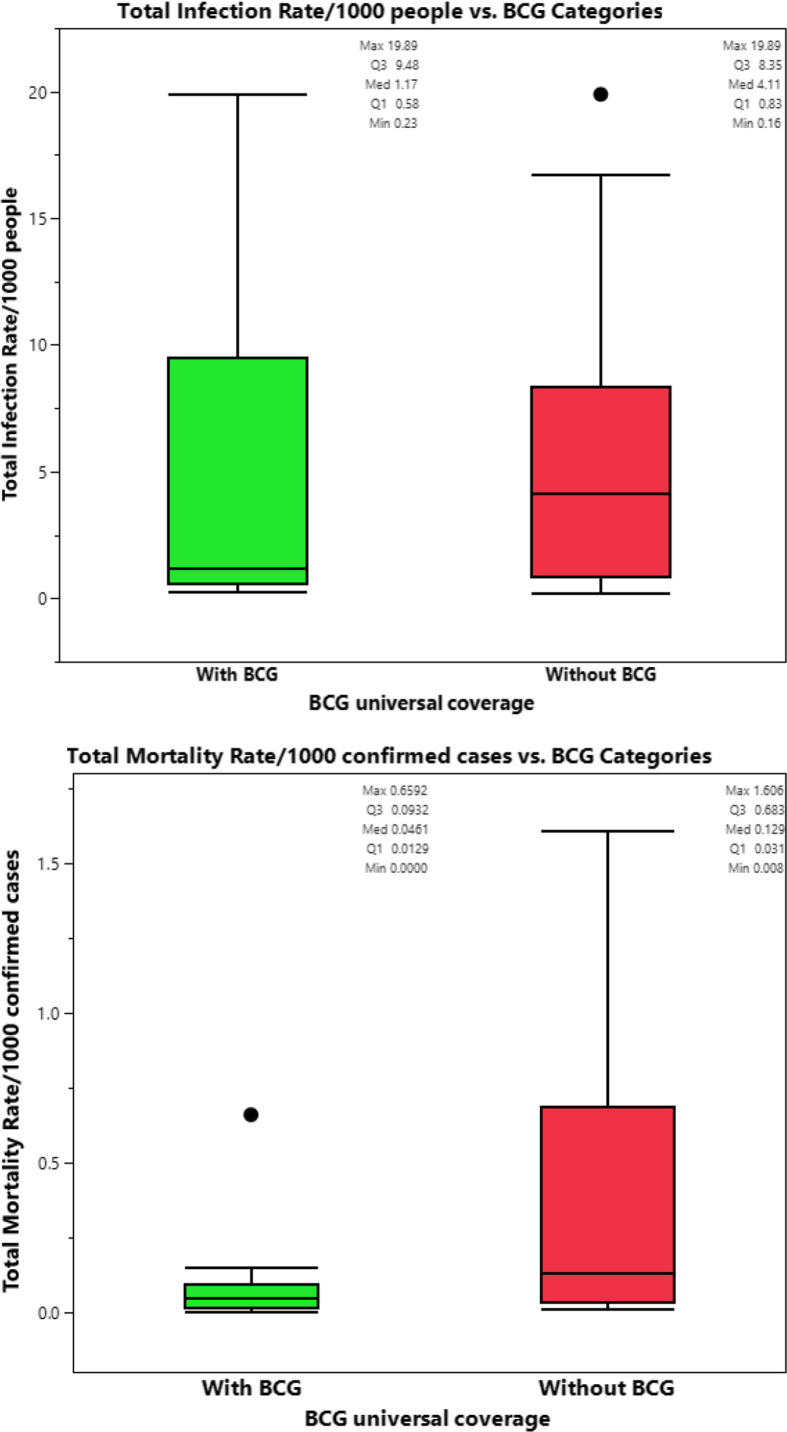
(a-b) Comparison of crude total infection rate/1000 people and total crude mortality rates/1000 confirmed cases between high-income countries with and without BCG universal coverage.

The prevalence of BCG vaccination coverage was significantly decreased with increasing income. The prevalence of vaccination coverage was 100%, 100%, 94.4%, and 38.2% in countries with lower income, lower middle income, upper middle income, and high income (*P* < 0.001), respectively. However, the prevalence of MCV2 was significantly increased with increasing income. The prevalence of MCV2 vaccination coverage was 58.1%, 84.8%, 88.9%, and 80.0% in countries with lower income, lower middle income, upper middle income, and high income (*P* = 0.005), respectively. A similar pattern of MCV2 was found for PCV3. The prevalence of PCV3 vaccination overage was 77.4%, 80.4%, 63.0%, and 90.9%, respectively (Table 5). Comparison of crude total mortality rates/1000 confirmed cases in countries with high-income economic status according to BCG vaccine coverage showed significant difference in (*P* = 0.005), but not in crude total infection rate/1000 people (*P* = 0.431), see Figure 6.

**Table 5.** Association of respiratory vaccination with status of economy

Vaccines	Economic status no. (%)				*P*-Value
	Low-income economies	Lower middle-income economies	Upper middle-income economies	High-income economies	
BCG categories					
With universal coverage vaccine	31 (100)	46 (100)	51 (94.4)	21 (38.2)	<0.001
Without universal coverage vaccine	0 (0.0)	0 (0.0)	3 (5.6)	34 (61.8)	
MCV2 categories					
With universal coverage vaccine	18 (58.1)	39 (84.8)	48 (88.9)	44 (80.0)	0.005
Without universal coverage vaccine	13 (41.9)	7 (15.2)	6 (11.1)	11 (20.0)	
PCV3 categories					
With universal coverage vaccine	24 (77.4)	37 (80.4)	34 (63.0)	50 (90.9)	0.006
Without universal coverage vaccine	7 (22.6)	9 (19.6)	20 (37.0)	5 (9.1)	

Note: Pearson chi-squared test was performed for statistical analyses. DTP1 and DTP3 were not included in this table due to having universal coverage of these vaccines in all countries, MCV1 (not practiced by only 1 country), PCV1 and PCV2, and PCV3 have same number of countries with and without universal vaccination coverage.

## Discussion

To our knowledge, this study is the first to present COVID-19 infection and mortality rates in the light of age at vaccination and vaccine years for respiratory infectious diseases. Herein, we demonstrate significant reductions in COVID-19 incidence and mortality rates with BCG vaccination and a paradoxical increase in COVID-19 mortality with widespread MCV2 and PCV3 years. Also, we found that DTP1, MCV1, PCV1, and PCV2 had no protective effect on the infection and mortality rates of COVID-19.

We found that the total infection and mortality rates of COVID-19 were increased with increasing income. But, this study showed that the high-income countries were more likely to not have BCG and to have MCV2 and PCV3 vaccination coverage. Indirectly, we can say that BCG vaccination has decreased and MCV2 and PCV3 have increased the infection and mortality rates of COVID-19.

BCG vaccine is known to influence immune host responses to numerous infections and has nonspecific effects that lead to reductions in infection and mortality. In animal studies, BCG protects against RNA and DNA viral pathogens and similar BCG-associated immune effects against viral illness have been demonstrated in humans as well.^15^ It is presumed that BCG induces immune memory by activating heterologous lymphocytes, resulting in modulation of cytokine production, macrophage activation, T-cell responses, and antibody titers.^15^

In two randomized controlled trials of BCG administration at birth versus delayed administration in low birth weight Danish infants, BCG vaccination at birth was associated with significant reductions in infectious disease-related mortality.^20,21^ Similarly, infants randomized to early BCG vaccination in Guinea-Bissau had reductions in all-cause lower respiratory tract hospitalization, including for respiratory syncytial virus.^22^ Other placebo-controlled trials of BCG vaccination have demonstrated more robust immunological responses to influenza vaccination,^23^ all-cause pneumonia,^24^ and human papillomavirus virus infection.^25^ The studies that have investigated the impact of BCG vaccination on respiratory infections have not performed the discrimination between respiratory tract infections caused by bacteria or viruses.^15^ In our study, we have demonstrated that universal BCG vaccination was associated with reducing COVID-19 infection and mortality.

Our data suggest that BCG-induced nonspecific immune effects against SARS-CoV-2 may be sufficient to modulate the infection of illness at a population level. In addition, it could further assist the patient to fight against the disease to reduce mortality. However, we did not find a significant association of infection and mortality rates with age at vaccination and BCG vaccine years in this study. The possible reason is that most of the countries administer the BCG vaccination at birth with sufficient coverage for many years. Therefore, it does not give us the accurate finding on the role of age at vaccination and BCG vaccine years on infection and mortality of the SARS-CoV-2.

A study^23^ included 40 healthy volunteers to receive live attenuated BCG vaccine (*n* = 20) or placebo (*n* = 20) in a randomized controlled trial. The healthy adults received an intramuscular injection of trivalent influenza vaccine after 14 d. The study reported that HI (hemagglutination inhibiting) antibody responses were significantly improved against the 2009 pandemic influenza A (H1N1) vaccine strain in BCG-vaccinated subjects. The nonspecific effects of influenza vaccination were observed along with enhanced pro-inflammatory leukocyte responses following BCG vaccination. The cytokine responses against nonrelated pathogens were modulated as well.^23^ The efficacy and safety effects of viable BCG vaccination on plane warts in children showed the complete response in 65% of children with common warts and 45% with plane warts in contrast with no response in the control group.^26^

Higgins et al.^27^ systematically reviewed the effect of BCG on all-cause childhood mortality and concluded that these vaccines prevent far more deaths than would be anticipated by coverage for the targeted diseases that they prevent. Nonspecific effects of vaccines against SARS-CoV-2 infection and mortality should be explored systematically once more data becomes available during the pandemic.

A recent study reviewed the current evidence of BCG vaccination in preventing severe infectious respiratory diseases other than tuberculosis (TB).^28^ They reported that the BCG vaccine has a strong protective effect against both upper and lower acute respiratory tract infections. They reported that the countries with universal BCG vaccination policy have a significantly lower incidence of COVID-19 compared with countries without universal BCG vaccination policy. In addition, the BCG vaccine has a protective role against infections such as influenza A virus, pandemic influenza (H1N1), and other acute respiratory tract infections. However, they call for an urgent need for further evidence through well-designed investigations to find out the role of BCG vaccination over time and across age groups.^28^

We found that countries with social insurance and social assistance health systems, and universal, social insurance, and social assistance systems have significantly higher infection and mortality rates of the COVID-19. We further understood that countries with social insurance and social assistance and universal, social insurance, and social assistance systems present health services based on the types of health insurance. In addition, social assistance has not covered all individuals across the country. Health insurance coverage has been reported to contribute to better health outcomes for adults. Moreover, it has been linked to access to a regular source of health care with greater and more convenient use of health services. But, we have no information about the experience of the adult population who do not seek health care, whether insured or uninsured. Especially, uninsured individuals are less likely to seek treatment. Furthermore, health insurance is more likely to improve health outcomes if it is continuous and links people to convenient care. Health insurance is more strongly linked to the receipt of suitable care when it includes preventive and screening services, prescribing medicines, and mental health care.^29^

Access to basic health services with acceptable quality is still not met in many countries in the world.^30^ Payments for health-care services are considered to be an obstacle to access to health care resulting in not-desirable outcomes, especially on equity.^31^ The socio-economic characteristics are important determinants for health and well-being. The people in lower socioeconomic groups have a greater risk for poor health and higher rates of illnesses, disability, and death.^32^ However, this study showed that countries with higher income were more likely to have higher total crude infection and mortality rates of COVID-19. This confirms that the lower-income countries have provided several health-care services, including immunization free of charge. Therefore, we attribute the higher infection and mortality rates of COVID-19 in high-income countries to immunity rather than the economy, because we found in our further analysis that higher-income countries are more likely to not receive the universal BCG vaccination and receive MCV2 and PCV3 vaccination coverage.

We found that the infection caused by COVID-19 was increased with increasing years of universal MCV2 vaccination. The WHO recommends that MCV2 be incorporated into the vaccination schedules of countries that have attained >80% MCV1 coverage for three consecutive years.^33^ Furthermore, the WHO recommends that countries seeking to eliminate measles should reach 95% coverage of MCV1 and MCV2 dosing, with both doses equitably administered to all children in every district. Administration of MCV2 in the second year of life decreases the pool of vulnerable children by eliciting targeted immunity in those who may not have responded to a single dose of the vaccine.^34^ Our data suggest that serial MCV doses may lead to the nonspecific weakening of the immune response to SARS-CoV-2.

We believe that more doses of a respiratory vaccine could decrease immunity rather than enhancing it. We call on further investigations on the effect of MCV2 and PCV3 on immunity parameters for respiratory diseases.

We found that countries with universal PCV1-3 vaccines coverage have significantly higher total mortality rates compared with those countries without these vaccines. This may indicate the importance of pneumococcus as a co-infecting pathogen in patients with COVID-19. In the context of influenza, pneumococcus is known to be an important contributor to disease severity and mortality,^35^ and in the context of COVID-19 pneumococcus has been identified as being an important co-infecting pathogen.^36^

The present study found that universal BCG vaccination, but not age at vaccination and vaccine years, was associated with reduced COVID-19 associated infection and mortality. This suggests that nonspecific effects of BCG may protect against the development of severe COVID-19, especially in countries with mature and established BCG vaccination coverage. Paradoxically, the length of universal MCV2 coverage was associated with an increase in COVID-19 associated infection in our study, which may reflect impaired immunity to SARS-CoV-2 in populations highly vaccinated with MCV. These effects need to be investigated further in future randomized controlled trials. We do not know the exact mechanism of the effects of PCV3 on infection and mortality because of respiratory diseases, but the emergence of PCV3 warrants further investigation.^37^ Recently, the WHO reported that “Vaccines against pneumonia, such as pneumococcal vaccine and Haemophilus influenza type B (Hib) vaccine, do not provide protection against the new coronavirus.”^38^ However, it did not refer to any evidence.

## Limitations of the Study

This study has some limitations. Although we included all countries with COVID-19 confirmed cases as of May 29, 2020, not all countries were included in the analysis because of a lack of immunization schedule. COVID-19 infection rates, and mortality rates, are crude estimates based on detected COVID-19 cases only and so could not account for the inevitable under-reporting of cases. Furthermore, the analysis was not stratified by serotype or strain of viral pathogen that could potentially be vaccinated against, and the interactions that these pathogens could have with SARS-CoV-2.

Importantly, it must be taken into account that this is an ecological study. Therefore, the findings obtained in this study may accompany some other confounders not solved in this study.

## Conclusions

In conclusion, we demonstrate that countries with universal BCG vaccination coverage have significantly reduced SARS-CoV-2 associated crude infection and mortality. Furthermore, countries with widespread MCV2 vaccination appear to have increased SARS-CoV-2 associated infection rates. This indicates that nonspecific vaccine effects and potential co-infection with bacterial pathogens could impact on the clinical severity of COVID-19 at a population level.

We recommend that the effect of BCG vaccination on infection and mortality rates of the COVID-19 be tested in countries without universal BCG vaccination.
